# Structural and Mechanical Improvements to Bone Are Strain Dependent with Axial Compression of the Tibia in Female C57BL/6 Mice

**DOI:** 10.1371/journal.pone.0130504

**Published:** 2015-06-26

**Authors:** Alycia G. Berman, Creasy A. Clauser, Caitlin Wunderlin, Max A. Hammond, Joseph M. Wallace

**Affiliations:** 1 Department of Biomedical Engineering, Indiana University-Purdue University at Indianapolis, Indianapolis, IN, United States of America; 2 Weldon School of Biomedical Engineering, Purdue University, West Lafayette, IN, United States of America; 3 Department of Orthopaedic Surgery, Indiana University School of Medicine, Indianapolis, IN, United States of America; Rensselaer Polytechnic Institute, UNITED STATES

## Abstract

Strain-induced adaption of bone has been well-studied in an axial loading model of the mouse tibia. However, most outcomes of these studies are restricted to changes in bone architecture and do not explore the mechanical implications of those changes. Herein, we studied both the mechanical and morphological adaptions of bone to three strain levels using a targeted tibial loading mouse model. We hypothesized that loading would increase bone architecture and improve cortical mechanical properties in a dose-dependent fashion. The right tibiae of female C57BL/6 mice (8 week old) were compressively loaded for 2 weeks to a maximum compressive force of 8.8N, 10.6N, or 12.4N (generating periosteal strains on the anteromedial region of the mid-diaphysis of 1700 με, 2050 με, or 2400 με as determined by a strain calibration), while the left limb served as an non-loaded control. Following loading, *ex vivo* analyses of bone architecture and cortical mechanical integrity were assessed by micro-computed tomography and 4-point bending. Results indicated that loading improved bone architecture in a dose-dependent manner and improved mechanical outcomes at 2050 με. Loading to 2050 με resulted in a strong and compelling formation response in both cortical and cancellous regions. In addition, both structural and tissue level strength and energy dissipation were positively impacted in the diaphysis. Loading to the highest strain level also resulted in rapid and robust formation of bone in both cortical and cancellous regions. However, these improvements came at the cost of a woven bone response in half of the animals. Loading to the lowest strain level had little effect on bone architecture and failed to impact structural- or tissue-level mechanical properties. Potential systemic effects were identified for trabecular bone volume fraction, and in the pre-yield region of the force-displacement and stress-strain curves. Future studies will focus on a moderate load level which was largely beneficial in terms of cortical/cancellous structure and cortical mechanical function.

## Introduction

Bone is a dynamic hierarchical material that spans several orders of magnitude in length scale [1]. Bone has been shown to adapt its chemical makeup and structural organization in response to mechanical stimulation across these hierarchical length scales. Rodent models are often used to study specific aspects of bone’s adaptive response to loading. Exercise models including jumping, swimming, and running have been used as effective loading models in mice and rats with well-documented effects on skeletal structure and function [2–6]. These models have the advantage of being simplistic in design and physiologically relevant. However, in addition to whole body systemic effects, they prevent having control over loading parameters such as load/strain stimulus, cyclic design, and orientation of loading. Direct loading of individual limbs overcomes these obstacles and provides a way to control all aspects of loading to focus on the mechanisms underlying a response to specific mechanical cues.

The ulnar loading model has been successfully used for more than 15 years to study the response to loading in mice and rats [7–9]. More recently, axial loading of the mouse tibia has become a well-accepted model and has provided insight into the effects of loading as a function of age [10–18], sex [19–21], inbred strain [22], disease [23–26], fracture healing [27,28], and load/strain level [29–33]. Interestingly, despite the increasing use of tibial loading in mice, few studies have placed significance on cortical mechanical outcomes. One study looked at mechanics in a fracture healing model, where the fracture site was directly loaded [28]. A second study performed axial loading to failure following a 6 week tibial loading experiment and reported an increase in structural mechanical properties [22]. A third study indirectly calculated elastic modulus by assuming a relationship between ash mineral density and the attenuation coefficient obtained from micro-computed tomography (μCT) [18].

The majority of studies have investigated the effect of tibial loading on bone formation using dynamic histomorphometry and/or μCT. These outcomes are important to assess how and where bone forms in response to loading. However, if changes in formation fail to improve functional properties of the bone, an increase in bone formation could be less compelling. For example, if more bone is formed due to loading, but that bone is not better equipped to bear load or resist fracture, loading did not achieve a beneficial functional outcome [11,22]. For this reason, it is important to investigate mechanical outcomes alongside formation.

To remain consistent with the majority of tibial loading studies, female C57BL/6 mice were used here. Although studies have used this strain of mice with starting ages ranging from 6 weeks to 22 months, we chose to use mice at 8 weeks of age to be consistent with previous studies in our lab utilizing treadmill running [34–36]. The goal of this study was to investigate the cortical mechanical implications of targeted *in vivo* loading in addition to changes in cortical and trabecular architecture. We hypothesized that loading in female mice would lead to dose-dependent increases in cortical and trabecular architectural parameters as well as increased mechanical stiffness, strength, and ductility in the diaphysis.

## Materials and Methods

### Animals

Animals (n = 35, female, C57BL/6NHsd) were obtained from Harlan Laboratories (Indianapolis, IN) at approximately 7 weeks of age and allowed to acclimate for one week prior to the start of *in vivo* loading. Animals were handled following Indiana University School of Science Institutional Animal Care and Use Committee (IACUC) approval (SC210R) and group-housed with access to food and water *ad libitum* in a light/dark controlled room. Body mass was recorded two days prior to the beginning of loading and animals were randomly sorted into weight-matched groups (three groups of 10 for loading, one group of 5 for calibration). Animals were weighed every other day after the start of loading to assess overall health.

### Strain Calibration

**Fig 1:**
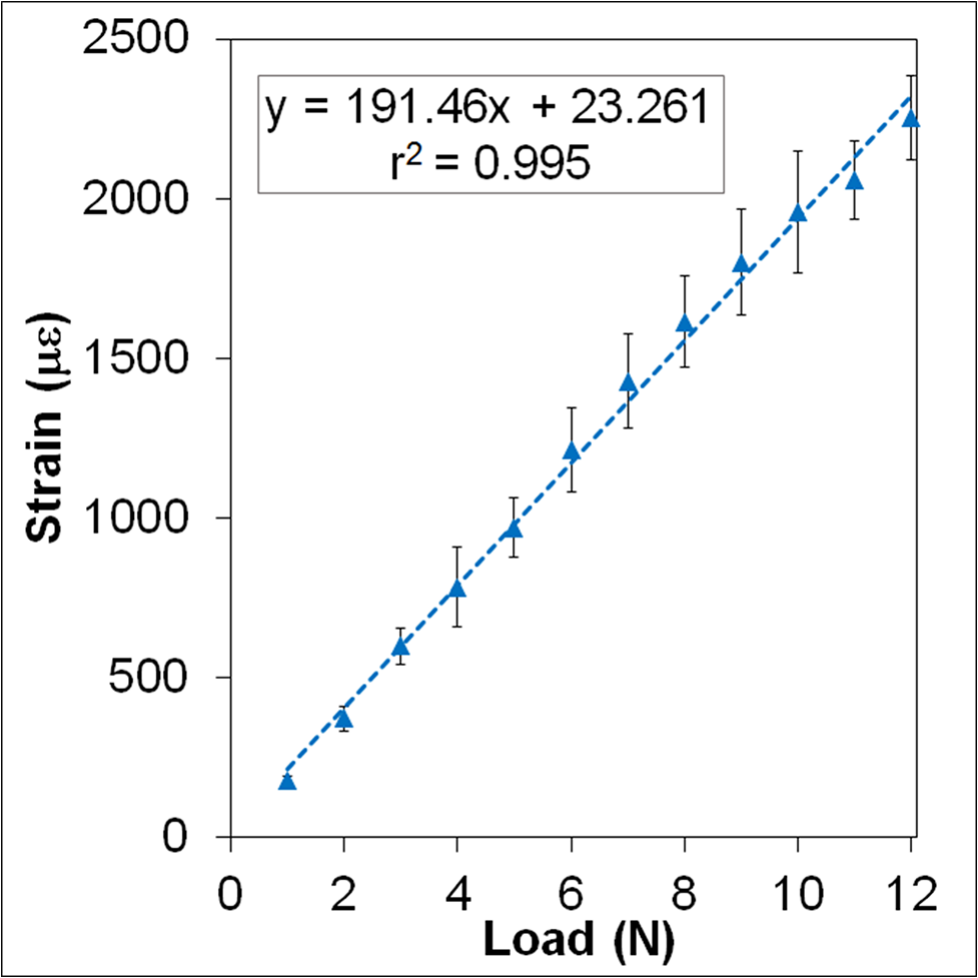
Load/Strain Calibration Curve. This figure demonstrates the linear relationship between applied load and resulting tensile strain at the periosteal mid-diaphysis. For all data points, error bars represent the standard deviation from n = 5 mice.

One day prior to beginning *in vivo* loading, five mice were sacrificed via CO_2_ inhalation. Immediate after sacrifice, a small incision was made through the skin of the right tibia and the skin was retracted in order to attach a single-element microstrain gauge (Vishay, Shelton, CT: EA-06-015DJ-120) to the anteromedial surface of the bone, proximal to the tibia-fibula junction. The exposed bone surface was first cleaned using chloroform and the gauge was attached using an adhesive kit (M-Bond 200). After briefly drying, the gauge was coated with polyurethane (M-Coat A) and the skin was released to cover the wound. Using a mechanical testing machine (Bose Corporation, Eden Prairie, MN: Electroforce 3200) equipped with a 45 N load cell and a custom loading fixture, the tibiae were loaded using a 2 Hz haversine waveform and load was stepped up from 2 N to 12 N in 1 N increments. Load and strain were recorded simultaneously. Load versus strain curves were plotted and a linear fit was applied to obtain the relationship between load and tensile strain (Fig 1). Using this calibration curve, it was determined that a maximum compressive force of 8.8N, 10.6N, or 12.4N was needed to generate periosteal mid-diaphyseal tensile strains of 1700 με, 2050 με, or 2400 με, respectively.

### *In vivo* Loading

**Fig 2:**
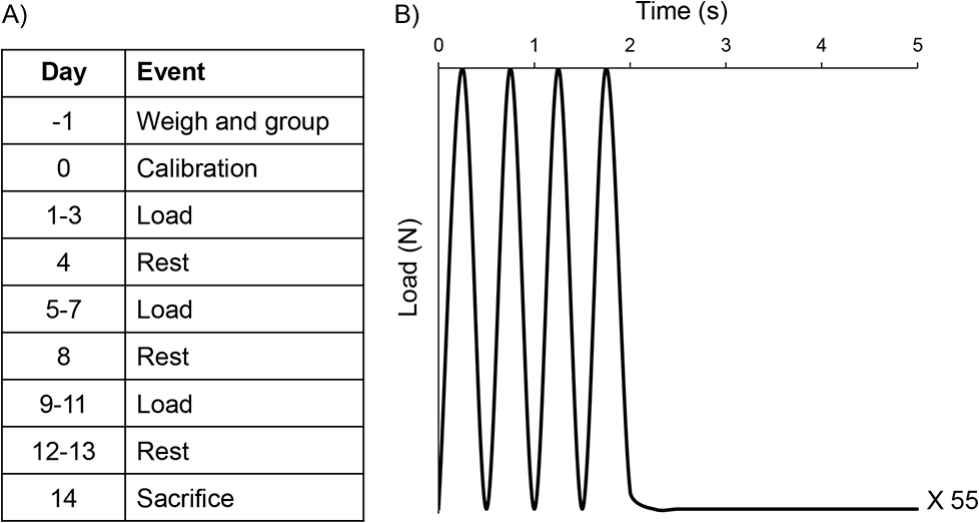
Timeline for tibial loading study and waveform profile. A) The right tibia of each mouse was loaded using the waveform to a set force (8.8N, 10.6N and 12.4N) to elicit a desired periosteal mid-diaphyseal tensile strain level (1700 με, 2050 με and 2400 με) over a 14 day period. B) The loading profile consisted of four haversine waveforms followed by 3 seconds of rest repeated 55 times for a total of 220 cycles of loading per day.

The three remaining groups of mice (n = 10 each) were randomly assigned to low strain, mid strain, and high strain loading. Isoflurane-induced anesthesia (2%) was used to anesthetize mice prior to loading and mice were maintained under anesthesia for the duration of loading. Right tibiae were loaded (cyclic compression, 2 Hz) over a 14 day period with a day of rest after every third day of loading, resulting in 9 loading days (Fig 2A). The loading profile consisted of four haversine waveforms to the maximum compressive load level noted above followed by 3 seconds of rest (at the max load level) repeated 55 times for a total of 220 cycles of loading per day (Fig 2B). After the final bout of loading, mice were allowed to rest for two days before sacrifice. Animals were euthanized at 10 weeks of age via CO_2_ inhalation. Right and left tibiae were harvested, total length was measured using calipers, and each bone was wrapped in phosphate buffered saline (PBS)-soaked gauze and stored at -20°C.

### Micro Computed Tomography (μCT)

All tibiae were thawed and then scanned using a high-resolution μCT system (Bruker-MicroCT, Kontich, Belgium; Skyscan 1172). Calibration was performed daily prior to scanning the bones using two cylindrical hydroxyapatite phantoms (0.25 and 0.75 g/cm^3^ CaHA). Scans were performed on hydrated bones with the long axis oriented vertically at an isotropic voxel size of 10.2 μm resolution (V = 60 kV, I = 167 μA), then reconstructed for use in cortical and trabecular analyses. After scanning, bones were wrapped in PBS-soaked gauze and stored at -20°C until mechanical testing.

**Fig 3:**
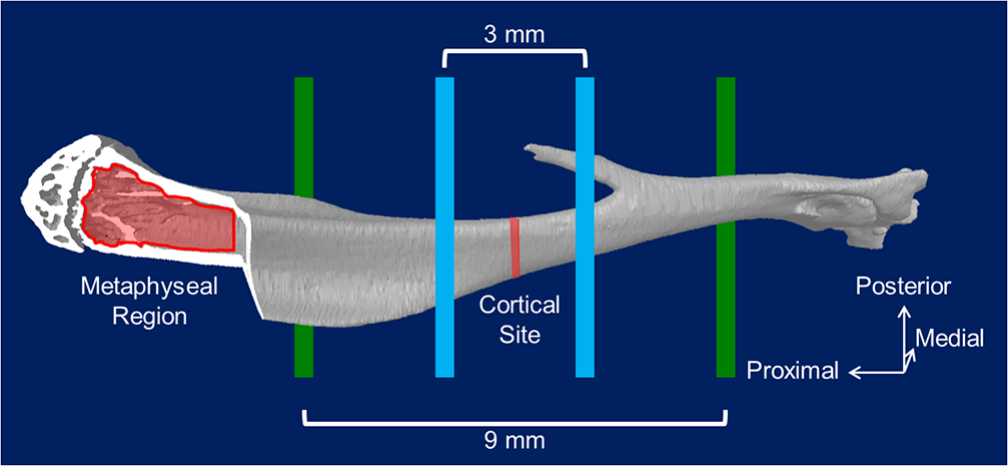
Location of mechanical tests and micro-computed tomography (μCT) regions of interest. The diaphysis of each bone was loaded using 4-point bending with the medial surface in tension. The bottom support points (green) were located 9mm apart and the top loading points (blue) were located 3mm apart. The metaphyseal region used for trabecular analyses began just distal to the growth plate in the proximal metaphysis and extended distally by 12% of the overall bone length. The cortical standard site was located 45% the total bone length from the proximal growth plate. Strain gauges used for calibration were in the region spanning the cortical standard site on the anteromedial surface.

For cortical bone analysis, a standard diaphyseal site was chosen 45% of the bone’s total length away from the proximal growth plate (Fig 3). Seven transverse slices were obtained from this site and tissue mineral density (TMD) was determined using vendor-supplied software (CTAn). The slices were then converted to binary images with a grayscale threshold value of 75. Cortical geometric properties were determined from these images using a custom code (MathWorks, Inc. Natick, MA, MATLAB). Calculated properties included areas (total cross sectional, cortical, and marrow), cortical thickness, widths (anterior-posterior [AP] and medial-lateral [ML]), perimeters (periosteal and endocortical), and principal moments of inertia.

Trabecular analysis was performed on 12% of the total bone length in the proximal metaphysis starting at the distal end of the growth plate. Regions of interest including only cancellous bone were automatically segmented using a custom Matlab code. Parameters of trabecular architecture (bone volume fraction [BV/TV], trabecular thickness, number, separation, and structure model index) and TMD were obtained using vendor-supplied software (CTAn).

### Mechanical Testing

Following μCT, all tibiae were thawed and then monotonically tested to failure using four-point bending in displacement control at 0.025 mm/sec while fully hydrated, as previously described [37]. A loading span of 3 mm and support span of 9 mm were used (Fig 3). The tibia-fibula junction was placed just outside of the right-most loading point and oriented such that the bone was tested in the medial-lateral direction with the medial surface in tension. The distance from the proximal end of the bone to the location of fracture initiation was measured using calipers. Seven transverse slices were obtained from μCT images at the location of fracture and the calculated geometric properties (bending moment of inertia and distance from the centroid to the extreme fiber in tension) were used to map load-displacement into stress-strain. Pre- and post-yield mechanical properties were obtained from the resulting curves, as previously described [37].

### Statistics

The assumptions of normality and homoscedasticity were assessed and any violations were corrected using transformations. A repeated-measures ANOVA tested the main effects of loading (within-subject effect), strain level (between-subject effect), and their interaction (α = 0.05). If strain level had a significant main effect in the absence of an interaction effect with loading, Tukey’s HSD tests examined pairwise differences between strain levels (α = 0.05). If an interaction was indicated, simple main effects were investigated using an appropriate model given the nature of the groups within each variable and a Bonferroni correction was applied as in previous reports [10,19]. Paired t-tests evaluated the effect of loading at each of the three strain levels (i.e. control limb versus loaded limb) and a one-way ANOVA evaluated the effect of strain level separately within the loaded and non-loaded limbs. After the Bonferroni correction, the significance level for the paired t-tests and one-way ANOVAs was set to α = 0.01. If strain level had a significant effect from the ANOVA in either limb, post hoc pairwise differences were examined using Tukey’s HSD tests in that limb (α = 0.01). For the comparison of body mass between the start and end of loading, a repeated-measures ANOVA tested the main effects of age (day -1 vs. day 14) and strain level. Statistical tests were performed using SAS 9.4 (SAS Institute Inc., Cary, NC) and values are reported as the mean ± the standard deviation (SD).

## Results

Following sacrifice, the tibiae were first scanned by μCT to analyze the structural adaptation of the bones. During the initial characterization of these scans, a woven cortical response was discovered in the loaded tibiae of five mice from the 2400 με group. The decision was made to remove both the loaded and non-loaded contralateral limbs from all future analyses as the inclusion of these data would grossly skew the results (μCT and mechanical), reducing the sample size in each 2400 με group to n = 5. Data from the woven bone response have been included in the supplemental information, although no statistics were performed with those data included.

### Animal Body Mass and Tibial Length

The body mass of each animal was recorded two days prior to the start of loading at Day -1 (1700 με: 18.8 ± 1.0 g; 2050 με: 18.8 ± 0.9g; 2400 με: 18.3 ± 1.1g) and again on the final day of loading (1700 με: 19.5 ± 1.2g; 2050 με: 19.2 ± 1.0g; 2400 με: 18.5 ± 1.3g). Body mass and tibial lengths from animals with woven bone response were excluded. There was a significant increase in mass due to age (p<0.001), but no difference in body mass between groups. Tibial length was recorded at the end of the study. The length was compared between the control (1700 με: 16.9 ± 0.6 mm; 2050 με: 17.3 ± 0.4 mm; 2400 με: 17.0 ± 0.3 mm) and loaded limbs (1700 με: 16.9 ± 0.5 mm; 2050 με: 17.2 ± 0.5mm; 2400 με: 17.0 ± 0.5mm). There was no main effect of loading or strain level on tibial length.

### Cortical and Cancellous Architecture

In a standard cancellous region of interest in the proximal metaphysis, all properties had main effects of loading, strain level, or interaction effects (Table 1). Trabecular thickness and TMD had significant interaction effects (p = 0.038 and p = 0.042 respectively), and post hoc paired t-tests indicated that both were significantly higher in the loaded versus control limb at all three strain levels (p<0.001 in all cases). Trabecular thickness also increased as a function of strain level in the loaded limbs (p<001). Bone volume fraction (BV/TV) had a significant main effect of strain level without a significant interactive effect (p = 0.027) indicating a systemic effect of loading, which was generally enhanced as strain increased. In addition to the effect of strain level, there was also a significant main effect of loading in BV/TV (p = 0.013) with an increasing trend in the loaded limb. There was a main effect of loading which increased trabecular separation (p = 0.012), decreased trabecular number (p = 0.001), and increased structure model index (p<0.001). These results suggest that loading caused fewer, thicker, and more rod-like trabeculae resulting in a greater bone volume in the metaphysis.

**Table 1:** Cancellous Architecture in the Proximal Metaphysis.

	1700 με		2050 με		2400 με	
	Control (n = 10)	Loaded (n = 10)	Control (n = 10)	Loaded (n = 10)	Control (n = 5)	Loaded (n = 5)
BV/TV (%) ^♯,*^	4.34 ± 1.14	4.66 ± 1.18	5.33 ± 0.86	5.30 ± 0.67	5.31 ± 0.80	6.33 ± 0.89^a^
Trabecular Thickness (μm) ^&^	51.0 ± 1.5	58.7 ± 2.9^†^	52.1 ± 1.2	61.4 ± 2.3^†^	51.2 ± 1.3	66.0 ± 3.8^†,c^
Trabecular Number (1/mm) ^♯^	0.85 ± 0.22	0.79 ± 0.20	1.02 ± 0.18	0.86 ± 0.11	1.04 ± 0.17	0.97 ± 0.18
Trabecular Separation (mm) ^♯^	0.36 ± 0.04	0.37 ± 0.04	0.34 ± 0.03	0.35 ± 0.02	0.33 ± 0.03	0.35 ± 0.04
Structure Model Index ^♯^	2.44 ± 0.12	2.62 ± 0.18	2.37 ± 0.11	2.64 ± 0.11	2.37 ± 0.08	2.61 ± 0.14
Tissue Mineral Density (g/cm^3^) ^&^	0.84 ± 0.02	0.87 ± 0.02^†^	0.83 ± 0.02	0.88 ± 0.02^†^	0.83 ± 0.02	0.89 ± 0.03^†^

Values are presented as mean ± standard deviation. In the property column, ♯ indicates a main effect of loading and * indicates a main effect of strain level from repeated measures ANOVA (p<0.05) with no interactive effect. For the main effect of strain level in BV/TV, a post hoc Tukey’s HSD test examined pairwise differences between strain levels [‘a’ indicates a difference versus 1700 με (p<0.05)]. When there was a main interactive effect (indicated by & in the property column), a paired t-test evaluated the effect of loading at each strain level † (indicates p<0.01 versus control) and a one-way ANOVA with post hoc Tukey’s HSD tests (when p<0.01) evaluated differences between strain levels within loaded limbs [‘c’ indicates a difference versus 1700 με (p<0.01)].

**Fig 4:**
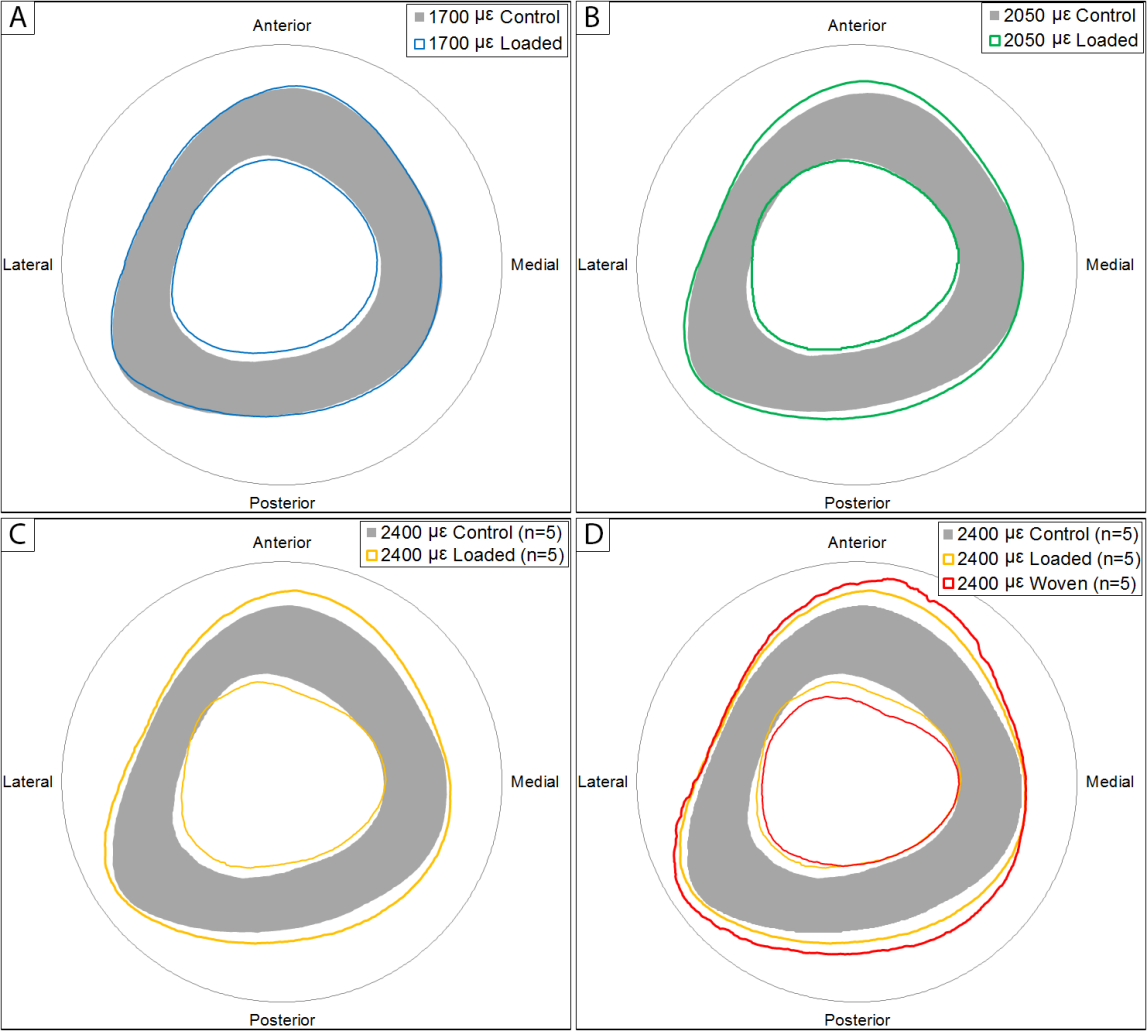
Schematic Representations of Standard Site Geometric Profiles. A) 1700 με group. B) 2050 με group. C) 2400 με group. There was a dose-dependent increase in cortical parameters with robust periosteal and endocortical formation, especially at the higher strain levels. D) 2400 με group with the addition of those animals that experiences a woven bone response due to loading.

Cortical geometry was analyzed at a standard site within the mid-diaphysis of each bone (Table 2, Fig 4). Strain level only affected the loaded limbs and there were no main effects of strain level indicating no systemic effects at this site. Generally, loading increased the amount of bone present in a dose-dependent fashion. Total cross-sectional area was increased with loading versus control in the 2400 με group (p<0.001). Cortical area and thickness were greater in the loaded limb at all strain levels (p<0.001 at all levels) and this effect was more pronounced as strain increased. Loading reduced marrow area and endocortical perimeter (main effect of loading, both p<0.001) indicating endocortical contraction. Loading increased maximum and minimum principal moments of inertia and, taking the increasing trend in periosteal perimeter into consideration (significant at 2400 με, p<0.001), these results indicate periosteal expansion accompanies the endocortical contraction. Periosteal expansion occurred primarily in the anterior-posterior (AP) direction as seen by significant increases in AP width as a function of strain in the loaded limb and the significant increase in loaded versus control limbs at 2050 με (p = 0.002), Medial-lateral (ML) width was unaffected by loading or strain level, as was cortical TMD.

**Table 2:** Cortical Geometry at the Mid-Diaphysis.

	1700 με		2050 με		2400 με	
	Control (n = 10)	Loaded (n = 10)	Control (n = 10)	Loaded (n = 10)	Control (n = 5)	Loaded (n = 5)
Cross Sectional Area (mm^2^) ^&^	0.836 ± 0.070	0.836 ± 0.050	0.827 ± 0.051	0.877 ± 0.033	0.833 ± 0.057	0.924 ± 0.019^†,d^
Cortical Area (mm^2^) ^&^	0.494 ± 0.042	0.524 ± 0.028^†^	0.496 ± 0.032	0.568 ± 0.027^†,c^	0.501 ± 0.036	0.628 ± 0.012^†,c,d^
Marrow Area (mm^2^) ^♯^	0.342 ± 0.033	0.311 ± 0.029	0.331 ± 0.023	0.310 ± 0.029	0.332 ± 0.026	0.296 ± 0.010
Cortical Thickness (mm) ^&^	0.186 ± 0.009	0.201 ± 0.007^†^	0.188 ± 0.007	0.215 ± 0.012^†,c^	0.189 ± 0.008	0.235 ± 0.004^†,c,d^
AP Width (mm) ^&^	1.050 ± 0.054	1.057 ± 0.030	1.016 ± 0.031	1.079 ± 0.024^†^	1.044 ± 0.052	1.127 ± 0.029^c^
ML Width (mm)	1.055 ± 0.050	1.053 ± 0.050	1.070 ± 0.056	1.080 ± 0.032	1.067 ± 0.038	1.104 ± 0.038
Periosteal Perimeter (mm) ^&^	3.885 ± 0.165	3.898 ± 0.113	3.858 ± 0.126	3.981 ± 0.070	3.888 ± 0.137	4.102 ± 0.046^†,d^
Endocortical Perimeter (mm) ^♯^	2.552 ± 0.122	2.449 ± 0.122	2.515 ± 0.094	2.431 ± 0.117	2.533 ± 0.102	2.401 ± 0.024
I_max_ (mm^4^) ^&^	0.055 ± 0.011	0.055 ± 0.008	0.054 ± 0.008	0.063 ± 0.006^†^	0.055 ± 0.009	0.072 ± 0.004^c^
I_min_ (mm^4^) ^&^	0.041 ± 0.006	0.043 ± 0.004	0.040 ± 0.004	0.047 ± 0.003^†^	0.041 ± 0.005	0.053 ± 0.003^†,c^
Tissue Mineral Density (g/cm^3^)	1.316 ± 0.023	1.328 ± 0.022	1.316 ± 0.021	1.318 ± 0.020	1.314 ± 0.024	1.301 ± 0.034

Values are presented as mean ± standard deviation. In the property column, ♯ indicates a main effect of loading from repeated measures ANOVA (p<0.05) with no interactive effect. When there was a main interactive effect (indicated by & in the property column), a paired t-test evaluated the effect of loading at each strain level († indicates p<0.01 versus control) and a one-way ANOVA with post hoc Tukey’s HSD tests (when p<0.01) evaluated differences between strain levels within loaded limbs [‘c’ indicates a difference versus 1700 με (p<0.01) and ‘d’ indicates a difference versus 2050 με (p<0.01)].

### Cortical Mechanical Properties from Four-point Bending

**Fig 5:**
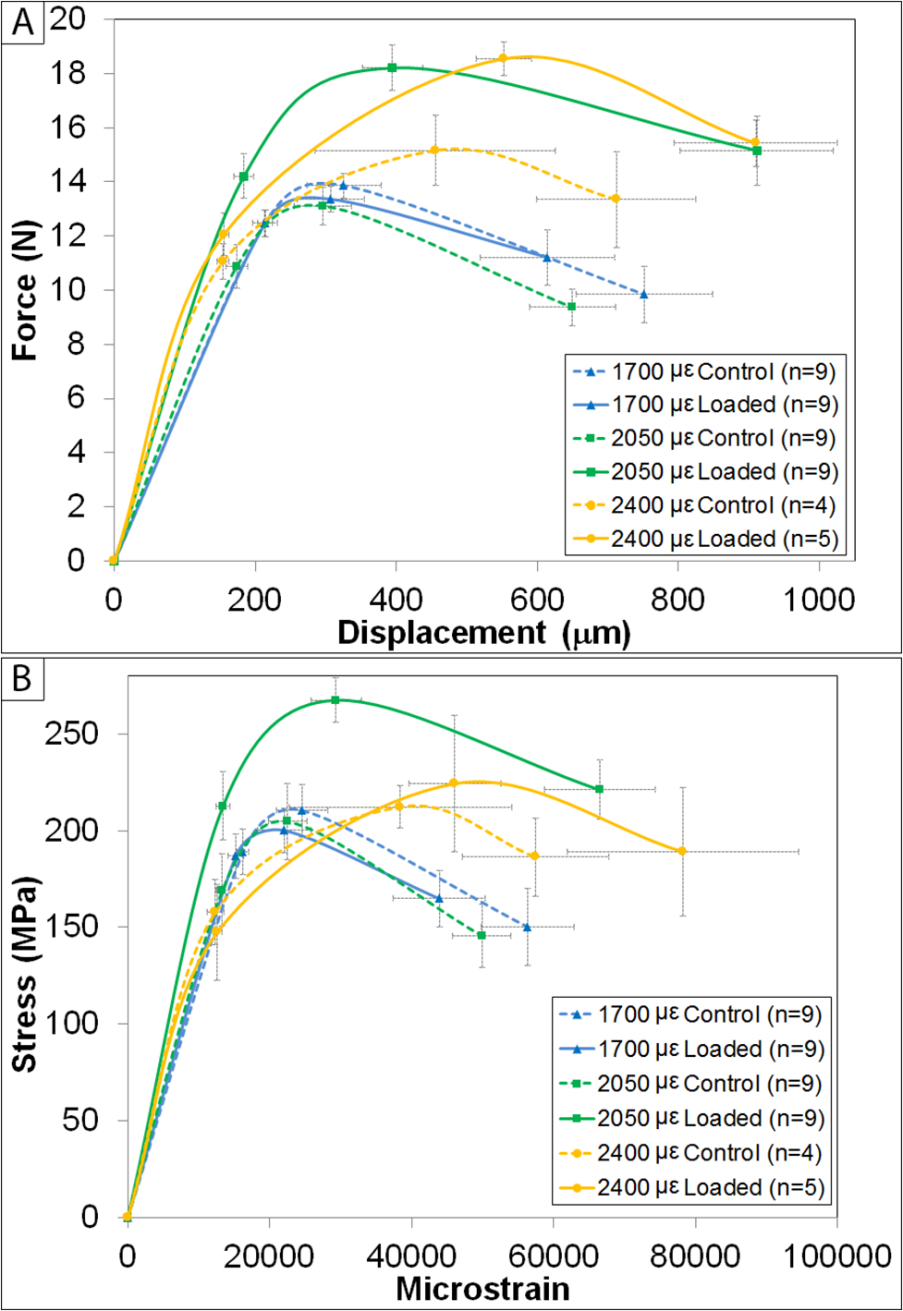
Schematic representations of mechanical testing curves. A) Representative structural-level force/displacement curves. B) Estimated tissue-level mechanical curves. At 1700 με, there was no mechanical effect noted. Those limbs loaded to 2050 με experienced significant increases in structural- and tissue-level strength and energy dissipation. The 2400 με group also experienced gains. However, when animals that experienced a woven bone response were removed from the analysis, the gains were more modest (especially at the tissue-level) and most failed to reach significance versus the contralateral control limb given the loss in power. For all data points, error bars represent the standard error of the mean (SEM).

Four-point bending was used to investigate changes in mechanical properties in the diaphysis due to *in vivo* loading. Structural and tissue-level mechanical properties are shown in Tables 3 and 4, respectively. In addition, schematic representations of mechanical data are shown in Fig 5. These schematic curves were generated by averaging data from the yield point, the point of maximum force/stress and the failure point across all samples within a group. Although they were not statistically analyzed in this form, the curves make a qualitative comparison between groups easier. As noted above, loaded and contralateral limbs from animals experiencing a woven bone response at the highest strain level were excluded from statistical analysis (data are shown in the supplemental information for the purpose of qualitative comparison). Excluding these bones dropped the sample size from n = 10 to n = 5 for the 2400 με group. In addition, the control group at that strain level had an excluded statistical outlier which, in the paired post hoc analyses, resulted in n = 4 for both the control and loaded groups.

**Table 3:** Structural Mechanical Properties From 4-Point Bending of the Mid-Diaphysis.

	1700 με		2050 με		2400 με	
	Control (n = 9)	Loaded (n = 9)	Control (n = 9)	Loaded (n = 9)	Control (n = 4)	Loaded (n = 5)
Yield Force (N) ^&^	12.47 ± 1.47	12.47 ± 1.46	10.88 ± 2.39	14.22 ± 2.48^‡^	11.05 ± 1.31	12.06 ± 1.73
Ultimate Force (N) ^&^	13.86 ± 1.39	13.36 ± 1.48	13.11 ± 2.11	18.21 ± 2.50^†,c^	15.16 ± 2.60	18.54 ± 1.37^c^
Stiffness (N/mm) ^*^	67.30 ± 13.05	70.80 ± 19.93	76.81 ± 16.53^a^	93.06 ± 12.60^a^	87.92 ± 8.76^a^	92.25 ± 9.82^a^
Deformation to Yield (μm) ^*^	214 ± 33	214 ± 51	174 ± 45^a^	184 ± 42^a^	154 ± 17^a^	155 ± 18^a^
Postyield Displacement (μm)	538 ± 292	401 ± 307	476 ± 174	727 ± 301	558 ± 226	755 ± 275
Total Deformation (μm)	752 ± 291	615 ± 286	650 ± 184	911 ± 326	712 ± 226	910 ± 259
Work to Yield (mJ)	1.45 ± 0.33	1.48 ± 0.38	1.06 ± 0.47	1.50 ± 0.58	0.96 ± 0.23	1.07 ± 0.26
Postyield Work (mJ) ^&^	5.81 ± 3.33	4.34 ± 3.30	4.96 ± 2.15	11.09 ± 4.29^†,c^	7.06 ± 2.72	11.95 ± 3.90^c^
Work to Failure (mJ) ^&^	7.26 ± 3.35	5.82 ± 3.20	6.02 ± 2.26	12.59 ± 4.60^†,c^	8.02 ± 2.76	13.01 ± 3.67^c^

Values are presented as mean ± standard deviation. In the property column, * indicates a main effect of strain level from repeated measures ANOVA (p<0.05) with no interactive effect. When there was a main effect of strain level, post hoc Tukey’s HSD tests examined pairwise differences between strain levels [‘a’ indicates a difference versus 1700 με (p<0.05)]. When there was a main interactive effect (indicated by & in the property column), a paired t-test evaluated the effect of loading at each of the strain levels († indicates p<0.01 versus control) and a one-way ANOVA with post hoc Tukey’s HSD tests (when p<0.01 which only occurred in loaded limbs) evaluated differences between strain levels within loaded limbs [‘c’ indicates a difference versus 1700 με (p<0.01)]. ‡ notes that for 2050 με, a marginal difference between loaded and control limbs for yield force (p = 0.02).

**Table 4:** Estimated Tissue-level Mechanical Properties From 4-Point Bending of the Mid-Diaphysis.

	1700 με		2050 με		2400 με	
	Control (n = 9)	Loaded (n = 9)	Control (n = 9)	Loaded (n = 9)	Control (n = 4)	Loaded (n = 5)
Yield Stress (MPa)	188.9 ± 35.4	186.8 ± 34.6	169.3 ± 56.0	212.6 ± 53.0	157.8 ± 33.7	147.5 ± 55.5
Ultimate Stress (MPa) ^&^	210.5± 40.7	200.1 ± 34.5	204.7 ± 59.0	267.5 ± 34.3^†^	212.3 ± 22.3	224.3 ± 79.2
Elastic Modulus (MPa)	13.83 ± 5.17	14.86 ± 5.27	15.61 ± 6.05	18.93 ± 3.96	16.69 ± 7.54	14.56 ± 7.14
Strain to Yield (με)^*^	16230 ± 2626	15221 ± 3724	13346 ± 2840^a^	13414 ± 2756^a^	12355 ± 2312^a^	12607 ± 1355^a^
Strain to Failure (με)^*^	56337 ± 19632	43864 ± 19630	49855 ± 12397	66456 ± 23364	57453 ± 20519	78167 ± 36418
Resilience (MPa)	1.64 ± 0.33	1.58 ± 0.45	1.26 ± 0.59	1.64 ± 0.68	1.07 ± 0.19	1.02 ± 0.32
Toughness (MPa) ^&^	8.32 ± 3.82	6.25 ± 3.69	7.11 ± 2.74	13.66 ± 5.29^‡,c^	8.87 ± 2.81	12.00 ± 2.20

Values are presented as mean ± standard deviation. In the property column, * indicates a main effect of strain from repeated measures ANOVA (p<0.05) with no interactive effect. When there was a main effect of strain level, post hoc Tukey’s HSD tests examined pairwise differences between strain levels [‘a’ indicates a difference versus 1700 με (p<0.05)].When there was a main interactive effect (indicated by & in the property column), a paired t-test evaluated the effect of loading at each of the strain levels († indicates p<0.01 versus control) and a one-way ANOVA with post hoc Tukey’s HSD tests (when p<0.01 which only occurred in loaded limbs) evaluated differences between strain levels within loaded limbs [‘c’ indicates a difference versus 1700 με (p<0.01)]. ‡ Notes that for 2050 με, a marginal difference between loaded and control limbs for toughness (p = 0.0123).

No structural or tissue-level mechanical properties in the 1700 με or 2400 με groups had a significant change with loading, but there were several significant differences within the 2050 με group. At 2050 με, loading significantly increased ultimate force (p<0.001), postyield work (p = 0.010), and work to failure (p = 0.010). There was also a trend towards increased yield force due to loading (p = 0.020). For estimated tissue-level properties, ultimate stress was significantly increased with loading in the 2050 με group (p = 0.007). In addition, although not significant given the stringent statistical threshold, toughness was marginally increased with loading (p = 0.012). Overall, the data indicate loading results in stronger tissue and structure which dissipates more energy prior to failure for the 2050 με group. Stiffness, deformation to yield, and strain to yield all exhibited systemic effects due to loading as indicated by the significant main effect of strain level (p = 0.007, p = 0.012, and p = 0.017, respectively). Strain to failure also had a significant main effect of strain (p = 0.050), but this effect was dominated by effects in the loaded limb.

## Discussion

Over the past 10 years, axial loading of the murine tibia has become a well-accepted and heavily utilized bone adaptation model. Despite this fact, few studies have explored the cortical mechanical impacts of targeted tibial loading [18,22,28] and instead have focused on morphological changes. While investigating an adaptive formation response and changes in bone architecture are important, failure to modify mechanical properties may lessen the broader impact of loading or the potential to use the model to enhance properties of diseased bone. The goal here was to investigate how cortical mechanical properties change in response to targeted tibial loading while also exploring changes in cortical and trabecular architecture. We hypothesized that loading would lead to dose-dependent increases in cortical and trabecular architecture while also increasing bone- and tissue-level stiffness, strength, and ductility in the diaphysis and, in general, data confirm this hypothesis. The different relationships between morphological and mechanical properties highlight the importance of assessing cortical mechanics along with morphology in future loading studies. Half of the mice loaded to 2400 με experienced a woven bone response. As discussed below, other factors indicated that loading to 2400 με in future studies should be avoided.

This study is not the first to investigate the strain-specific response to loading [30–33]. However, because the age and loading parameters in the various studies differ, there is no way to directly compare the responses. In addition, mechanical outcomes were rarely assessed. One study using female B6 mice at the same starting age as in our study (8 weeks) subjected the animals to load levels ranging from 5–13 N [31], but calibrated strain values were only reported for 12 week old mice so it is not clear what strain level these loads equated to at 8 weeks. In that study, loading tended to increase trabecular and cortical parameters at all ages with greater responses at higher load levels. A similar positive response to loading was reported for female B6 mice loaded between 0 and 14 N [32], but this group also reported a woven response in cortical bone at 14 N, similar to what was seen at 12.4 N in the current study. A third study loaded 26 week old female B6 mice to 5.9 N (1200 με) or 11.3 N (2100 με) for 2 weeks [30]. In that case, adaptation only occurred at the higher strain level where cancellous bone mass (via increased trabecular thickness) and cortical area (via periosteal and endocortical apposition) both increased. As with this previous work, a dose-dependent change in response to loading was noted in the current study for trabecular and cortical morphological parameters.

Improvements in trabecular architecture were driven by increases in trabecular thickness but at the cost of reducing trabecular number (Table 1). The reduction in trabecular number accounts for bone volume fraction only being significantly improved in the high strain group of the loaded limbs despite gains in thickness at all strain levels. Because the proximal metaphysis of this age and strain of mice is sparsely filled with trabeculae and continually declines with age starting at 8 weeks [38,39], small changes in the total number of trabecular struts could produce the significant decrease noted here. However, the decrease in the number of trabeculae may be an artifact of adjacent trabeculae thickening in the loaded limb to the point of closing their gap below the resolution of the μCT (~ 10 μm) and being tallied as one trabecular strut.

As in previous studies [22,40], neither cancellous nor cortical architecture displayed any systemic effects. In contrast, BV/TV was increased as a function of strain level in both loaded and control limbs. However, in the present study, both structural- and tissue-level mechanical properties were altered in the non-loaded limb as a function of strain level, particularly in the pre-yield region, indicating systemic effects. Loading to increasing strain levels systemically increased stiffness (~30% for non-loaded and loaded limbs from 1700 με to 2400με) causing bones to reach yield at lower displacements (Table 3) and strains (Table 4). While the main effect of strain level on elastic modulus did not reach significance, there was a general trend for increased elastic modulus at increasing strains, especially in the non-loaded limbs. A previous study showed no systemic changes in stiffness [22], but differences between these studies may be explained by age (22 weeks vs 10 weeks at sacrifice), duration of loading (6 weeks vs 2 weeks), mechanical testing modality (axial compression vs four-point bending), or strain level (compressive posterolateral 2800 με vs tensile anteromedial 2050 με) which, in the previous report, was high enough to damage existing tissue resulting in reduced stiffness. A caveat to this observation is that there was no *in vivo* imaging of bone structure in the mice at the start of the experiment. Therefore, despite our effort to randomize mice to groups through weight-matching, there is no way to verify a lack of enrollment bias in the morphology of the mice assigned to the different groups. Despite this limitation, the implications that loading could improve the mechanical integrity of existing tissue in the contralateral limb are intriguing. While it might not always be practical or possible to include a separate non-loaded cage control group in a study (e.g. in diseased mice where getting adequate sample sizes is challenging), these systemic effects should be considered in future studies investigating mechanical properties or mineralization.

The current study is also not the first to characterize cortical mechanical implications following tibial loading [18,22,28]. Only one previous study performed a full mechanical characterization following loading, but only reported structural properties by testing bones to failure in axial compression [22]. Given that the *in vivo* loading model is performed in axial compression, assessing *ex vivo* mechanical properties in this configuration is ideal since one would expect the adaptive response (if any) to be strongest in that orientation. In a pilot study, we attempted to load bones to failure in this manner but most failed at the epiphysis rather than in the diaphysis due to the compressive/bending loads induced in the bone. It is possible that because the animals used in the previous study were skeletally mature [22] versus the growing mice used here, the epiphyses were closed (or more fully mineralized) and therefore more structurally sound. Loading generally increased deformation and energy dissipation when bones were loaded to failure in axial compression [22]. In the current study, structural- and tissue-level energy dissipation were also increased, and although total deformation and strain to failure trended up, they failed to reach significance in any group. The current study also demonstrated increased ultimate force and ultimate stress with loading (Tables 3 and 4, Fig 5). These significant mechanical changes came specifically in the mid strain (2050 με) group. The lowest strain level (1700 με of tension) had little effect on structural and tissue-level strength and energy dissipation. The highest strain group (2400 με of tension) showed little to no increases in strength and energy dissipation versus those realized in the 2050 με group. Loading’s effect size (loaded versus control) at 2400 με was reduced due to values in the non-loaded limb trending toward the loaded values compared to the effect at 2050 με. Therefore, the lower sample sizes, only modest gains or losses in the loaded limbs, and baseline drift in the non-loaded limbs all contributed to the effect of loading at 2400 με not reaching significance.

Half of the animals loaded to 2400 με experienced a robust woven bone response, suggesting a potentially pathological response to loading (S1 and S2 Figs, S1 Table). It is important to note that many of the mice in this high strain group demonstrated a slight limping after recovering from anesthesia immediately following loading. The limp was short lived, but suggests that the loading itself may have been painful as previously shown when loading to 13 N [31]. When harvesting tissues, it was clear that some of the tibiae from the high strain group had bumpy nodules near the proximal end of the bone. These could potentially be the starting stages of osteophyte formation as recently demonstrated following high-magnitude mechanical loading [41]; however, further analysis of these nodules was not performed. When the woven response was discovered in five of these animals upon analysis of cortical μCT data, the decision was made to remove those animals from all analyses (both the loaded and its contralateral limb). The contralateral limb was removed as there was a concern of a potentially biasing a systemic response to loading. The data from the removed animals appear in the supplemental information for comparison but was not included in any statistical analyses. It is clear both from the mechanical data and to a lesser degree from the cortical and trabecular analysis that a woven response on the loaded side did in fact alter the non-loaded control limbs. While we believe this was the proper way to handle the data, the drawback of this decision was a loss of power. Our sample size started at n = 10 but the woven response dropped this to n = 5. The sample size was further reduced for the mechanical characterizations due to mechanical testing anomalies (one mechanical sample was lost from each group except the loaded bones of the 2400 με group). Because of the paired nature of the post hoc comparisons, this further reduces the sample size for the comparison of loaded versus control limbs (n = 8 for 1700 με and 2050 με; n = 4 for 2400 με). Although the starting sample size was sufficient for mechanical studies, losing samples and the associated lost power reduced our ability to detect differences in some groups. Therefore, despite what appear to be morphological and mechanical benefits, the combination of pain, a woven cortical response, and potential pathological nodule formation suggests that loading above the 2050 με level is unnecessary and should be avoided.

In conclusion, the current study demonstrated the expected positive impact of direct loading on cortical and cancellous architecture while also indicating that this form of loading, even in a short-term model, can lead to significant increases in structural- and tissue-level mechanical behavior in the diaphysis. This focus on mechanical end points is lacking in the literature but is important if the loading regimen is to used be for functional gain (e.g. to enhance properties of diseased bone). A woven cortical response initiated at the highest load level (12.4N, 2400 με) resulted in robust tissue formation and mechanical gains, but came at the cost of animal discomfort and a potential systemic response in the contralateral limb. Future studies utilizing this model should focus on a more moderate load level which was largely beneficial in young female mice both in terms of cortical/cancellous structure and cortical mechanical function.

## Supporting Information

S1 Fig Schematic representations of Standard Site Geometric Profiles from the 2400 με group. There is a potential systemic response when woven bone formation was initiated due to loading. The shaded bone in the background is from the control limb of animals with a normal formation response on the loaded contralateral side (solid yellow profile). When animals experienced a woven bone response due to loading (outermost periosteal and innermost endocortical profiles in red), the contralateral non-loaded limb (black) also appears to have experienced a primarily periosteal response. As a point of comparison, the periosteal perimeter of the non-loaded limb of animals experiencing a woven response increased by 0.78% versus the non-loaded limb of animals without a woven response. In the 1700 με group, the effect of loading was to increase the total cross sectional area by 0.34%.
(TIF)

S2 Fig Schematic representations of mechanical testing curves from the 2400 με group. This figure shows the systemic response when woven bone formation was initiated due to loading. The contralateral limb from animals which experienced a cortical woven bone response (black, lowest curve in each panel) had decreased strength and stiffness relative to the control limb from animals with no woven response (grey dashed curve). For all data points, error bars represent the standard error of the mean (SEM).
(TIF)

S1 Table Proximal Tibia Cancellous Architecture from the 2400 με Group. Values are presented as mean ± standard deviation. As opposed to the cortical and mechanical systemic response due to woven bone formation, the changes in cancellous bone were less pronounced.
(DOCX)
