# Supplementary figures and images for: Structural and Mechanical Improvements to Bone Are Strain Dependent with Axial Compression of the Tibia in Female C57BL/6 Mice
Source: PLoS One. 2015 Jun 26;10(6):e0130504. doi: 10.1371/journal.pone.0130504 (PMC4482632; doi:10.1371/journal.pone.0130504)

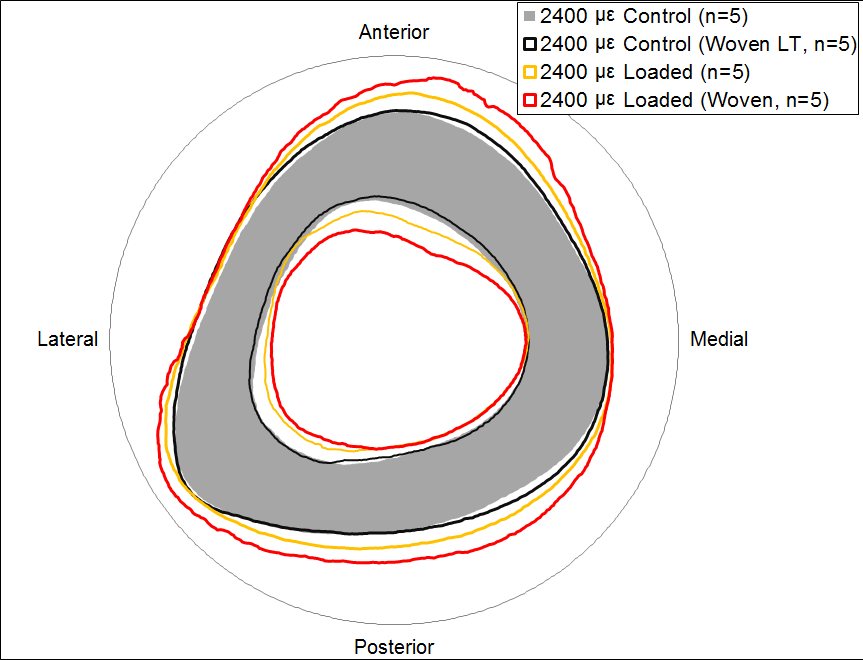

Supplement: S1 Fig — There is a potential systemic response when woven bone formation was initiated due to loading. The shaded bone in the background is from the control limb of animals with a normal formation response on the loaded contralateral side (solid yellow profile). When animals experienced a woven bone response due to loading (outermost periosteal and innermost endocortical profiles in red), the contralateral non-loaded limb (black) also appears to have experienced a primarily periosteal response. As a point of comparison, the periosteal perimeter of the non-loaded limb of animals experiencing a woven response increased by 0.78% versus the non-loaded limb of animals without a woven response. In the 1700 με group, the effect of loading was to increase the total cross sectional area by 0.34%. (TIF) [file pone.0130504.s001.tif]

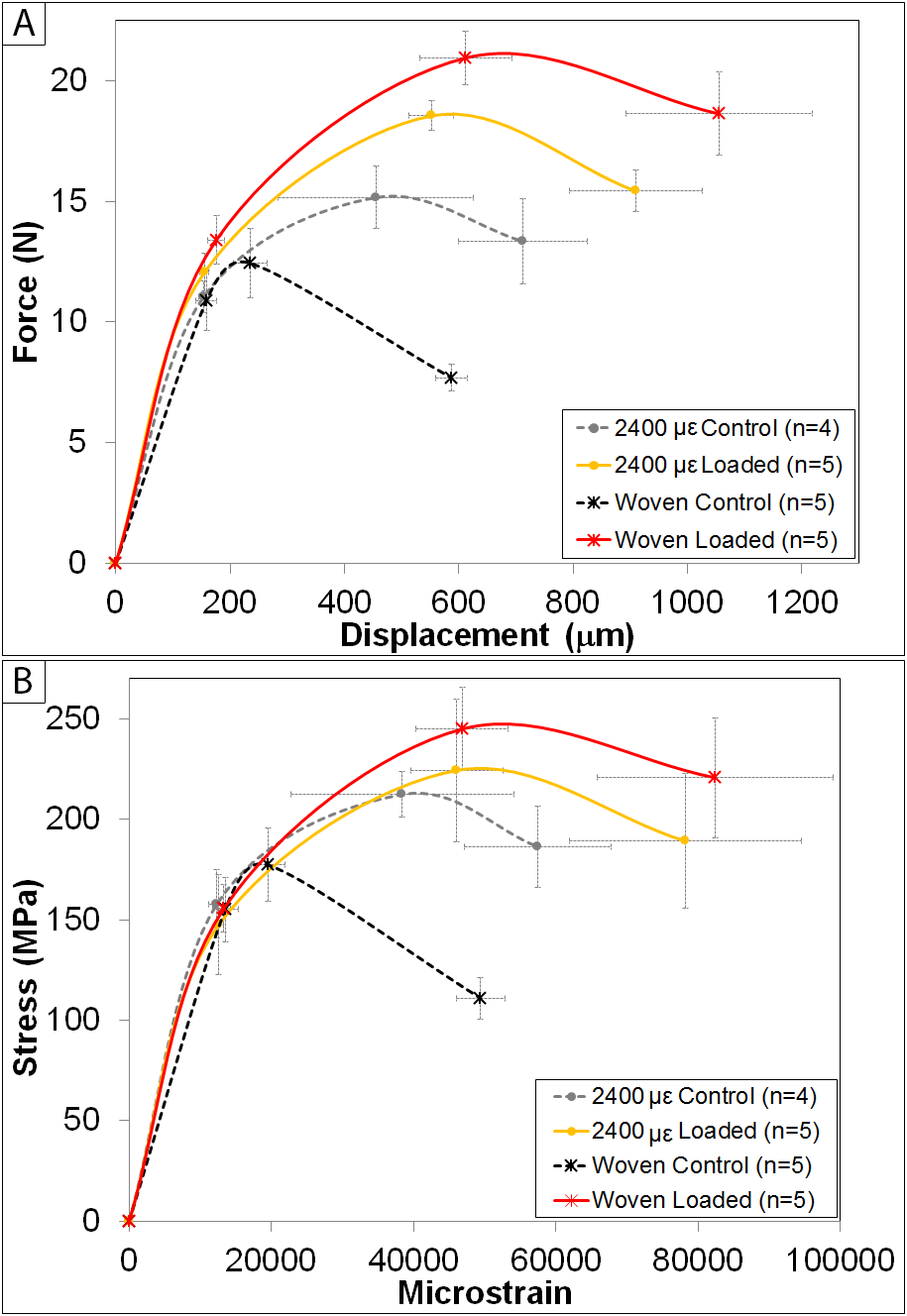

Supplement: S2 Fig — This figure shows the systemic response when woven bone formation was initiated due to loading. The contralateral limb from animals which experienced a cortical woven bone response (black, lowest curve in each panel) had decreased strength and stiffness relative to the control limb from animals with no woven response (grey dashed curve). For all data points, error bars represent the standard error of the mean (SEM). (TIF) [file pone.0130504.s002.tif]
