# Supplementary material for: Structural and Mechanical Improvements to Bone Are Strain Dependent with Axial Compression of the Tibia in Female C57BL/6 Mice
Source: PLoS One. 2015 Jun 26;10(6):e0130504. doi: 10.1371/journal.pone.0130504 (PMC4482632; doi:10.1371/journal.pone.0130504)
Supplement: S1 Table — Values are presented as mean ± standard deviation. As opposed to the cortical and mechanical systemic response due to woven bone formation, the changes in cancellous bone were less pronounced. (DOCX) [file pone.0130504.s003.docx]

**S1 Table. Proximal Tibia Cancellous Architecture from the 2400 με Group.**

|  | **Control (n=5)** | **Loaded (n=5)** | **Control from Woven Response (n=5)** | **Woven Response (n=5)** |
| --- | --- | --- | --- | --- |
| BV/TV (%) | 5.31 ± 0.80 | 6.33 ± 0.89 | 5.22 ± 0.93 | 6.39 ± 0.82 |
| Trabecular Thickness (μm) | 51.2 ± 1.3 | 66.0 ± 3.8 | 51.7 ± 1.7 | 72.6 ± 4.4 |
| Trabecular Number (1/mm) | 1.04 ± 0.17 | 0.97 ± 0.18 | 1.01 ± 0.16 | 0.88 ± 0.07 |
| Trabecular Separation (mm) | 0.33 ± 0.03 | 0.34 ± 0.04 | 0.35 ± 0.02 | 0.37 ± 0.03 |
| Structural Model Index | 2.37 ± 0.08 | 2.61 ± 0.14 | 2.33 ± 0.11 | 2.65 ± 0.05 |
| Tissue Mineral Density (g/cm^3^) | 0.83 ± 0.02 | 0.89 ± 0.03 | 0.85 ± 0.01 | 0.92 ± 0.03 |
| Values are presented as mean ± standard deviation. As opposed to the cortical and mechanical systemic response due to woven bone formation, the changes in cancellous bone were less pronounced. | | | | |
